# 1-[5-(4-Bromo­phen­yl)-3-(4-fluoro­phen­yl)-4,5-dihydro-1*H*-pyrazol-1-yl]ethanone

**DOI:** 10.1107/S1600536812033351

**Published:** 2012-07-28

**Authors:** Hoong-Kun Fun, Wan-Sin Loh, M. Sapnakumari, B. Narayana, B. K. Sarojini

**Affiliations:** a X-ray Crystallography Unit, School of Physics, Universiti Sains Malaysia, 11800 USM, Penang, Malaysia; b Department of Studies in Chemistry, Mangalore University, Mangalagangotri 574 199, India; c Department of Chemistry, P.A. College of Engineering, Nadupadavu, Mangalore 574 153, India

## Abstract

In the title mol­ecule, C_17_H_14_BrFN_2_O, the benzene rings form dihedral angles of 6.58 (6) and 85.31 (6)° with the mean plane of the 4,5-dihydro-1*H*-pyrazole ring (r.m.s. deviation = 0.0231 Å). The latter ring is planar with a maximum deviation of 0.032 (1) Å The dihedral angle between the benzene rings is 78.75 (6)°. In the crystal, weak C—H⋯O and C—H⋯F hydrogen bonds link the mol­ecules into corrugated layers parallel to the *ab* plane.

## Related literature

For our work on the synthesis of pyrazoline derivatives, see: Samshuddin *et al.* (2011 ▶). For related structures, see: Fun *et al.* (2010 ▶, 2012 ▶). For the stability of the temperature controller used in the data collection, see: Cosier & Glazer (1986 ▶).
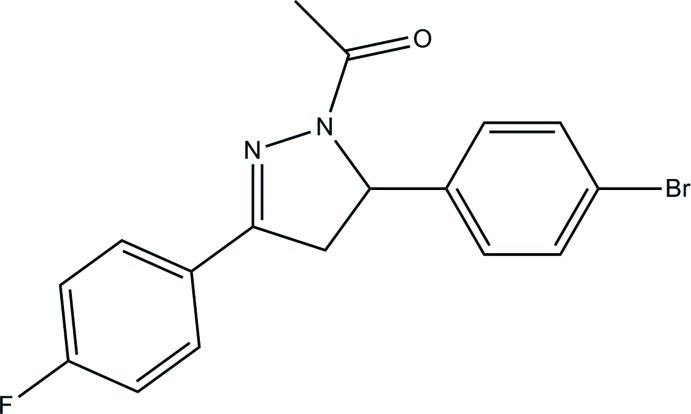


## Experimental

### 

#### Crystal data

C_17_H_14_BrFN_2_O
*M* *_r_* = 361.21
Monoclinic, 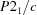

*a* = 6.0973 (5) Å
*b* = 12.3079 (11) Å
*c* = 20.1432 (16) Å
β = 96.700 (1)°
*V* = 1501.3 (2) Å^3^
*Z* = 4
Mo *K*α radiation
μ = 2.75 mm^−1^
*T* = 100 K
0.35 × 0.29 × 0.12 mm

#### Data collection

Bruker SMART APEXII DUO CCD area-detector diffractometer
Absorption correction: multi-scan (*SADABS*; Bruker, 2009 ▶) *T*_min_ = 0.449, *T*_max_ = 0.735
20560 measured reflections
5389 independent reflections
4508 reflections with *I* > 2σ(*I*)
*R*_int_ = 0.026

#### Refinement

*R*[*F* ^2^ > 2σ(*F* ^2^)] = 0.026
*wR*(*F* ^2^) = 0.064
*S* = 1.04
5389 reflections
200 parameters
H-atom parameters constrained
Δρ_max_ = 0.50 e Å^−3^
Δρ_min_ = −0.25 e Å^−3^

### 

Data collection: *APEX2* (Bruker, 2009 ▶); cell refinement: *SAINT* (Bruker, 2009 ▶); data reduction: *SAINT*; program(s) used to solve structure: *SHELXTL* (Sheldrick, 2008 ▶); program(s) used to refine structure: *SHELXTL*; molecular graphics: *SHELXTL*; software used to prepare material for publication: *SHELXTL* and *PLATON* (Spek, 2009 ▶).

## Supplementary Material

Crystal structure: contains datablock(s) global, I. DOI: 10.1107/S1600536812033351/cv5325sup1.cif

Structure factors: contains datablock(s) I. DOI: 10.1107/S1600536812033351/cv5325Isup2.hkl

Supplementary material file. DOI: 10.1107/S1600536812033351/cv5325Isup3.cml

Additional supplementary materials:  crystallographic information; 3D view; checkCIF report

## Figures and Tables

**Table 1:** Hydrogen-bond geometry (Å, °)

*D*—H⋯*A*	*D*—H	H⋯*A*	*D*⋯*A*	*D*—H⋯*A*
C4—H4*A*⋯O1^i^	0.95	2.45	3.2772 (15)	146
C14—H14*A*⋯F1^ii^	0.95	2.50	3.3806 (15)	153
C15—H15*A*⋯O1^iii^	0.95	2.45	3.3800 (15)	166

Symmetry codes: (i) 
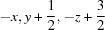
; (ii) 
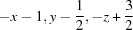
; (iii) 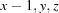.

**Fig. 1.**
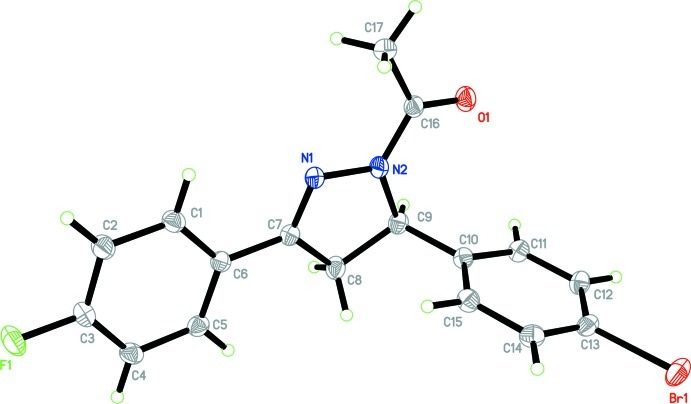
The molecular structure of the title compound, showing 50% probability displacement ellipsoids.

**Fig. 2.**
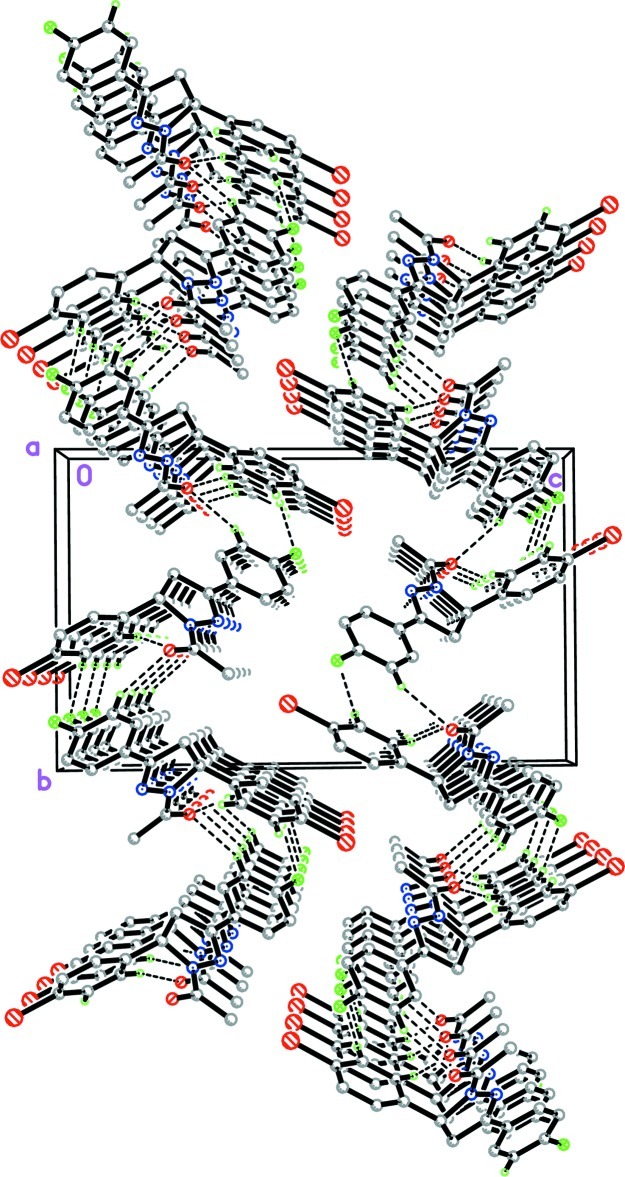
A portion of the crystal packing viewed along the a axis. H atoms not involved in the intermolecular interactions (dashed lines) have been omitted for clarity.

## supplementary crystallographic information

### Comment

In continuation of our work on the synthesis of pyrazoline derivatives (Fun *et al.*, 2010; Samshuddin *et al.*, 2011), the title compound is prepared and its crystal structure is reported.

In the title compound (Fig. 1), the two benzene rings (C1–C6 & C10–C15) form dihedral angles of 6.58 (6) and 85.31 (6)°, respectively, with the mean plane of 4,5-dihydro-1*H*-pyrazole ring (N1/N2/C7–C9, r.m.s. deviation = 0.0231 Å). The dihedral angle between the two benzene rings is 78.75 (6)°. Bond lengths and angles are comparable with those in the related structures (Fun *et al.*, 2010, 2012).

In the crystal packing (Fig. 2), intermolecular C—H···O and C—H···F hydrogen bonds (Table 1) link the molecules into corrugated layers parallel to the *ab* plane.

### Experimental

A mixture of (2*E*)-3-(4-bromophenyl)-1-(4-fluorophenyl)prop-2-en-1-one (3.05 g, 0.01 mol) and hydrazine hydrate (0.48 ml, 0.01 mol) in 30 ml acetic acid was refluxed for 6 h. The reaction mixture was cooled and poured into 50 ml ice-cold water. The precipitate was collected by filtration and purified by recrystallization from ethanol. The single-crystal was grown from acetone by slow evaporation method. *M.p.*: 372–374 K.

### Refinement

All the H atoms were located geometrically and were refined using a riding model with *U*_iso_(H) = 1.2 or 1.5 *U*_eq_(C) [C–H = 0.95 to 1.00 Å]. A rotating group model was applied to the methyl group. In the final refinement, ten outliners were omitted, namely - *2 0 8, 1 4 2, 1 0 4, -2 7 5, -4 2 9, -3 4 9, -1 8 1, -2 2 4, 1 8 0 and 1 1 4* , respectively.

### Crystal data

**Table:**

C_17_H_14_BrFN_2_O	*F*(000) = 728
*M**_r_* = 361.21	*D*_x_ = 1.598 Mg m^−^^3^
Monoclinic, *P*2_1_/*c*	Mo *K*α radiation, λ = 0.71073 Å
Hall symbol: -P 2ybc	Cell parameters from 7995 reflections
*a* = 6.0973 (5) Å	θ = 3.3–32.4°
*b* = 12.3079 (11) Å	µ = 2.75 mm^−^^1^
*c* = 20.1432 (16) Å	*T* = 100 K
β = 96.700 (1)°	Block, colourless
*V* = 1501.3 (2) Å^3^	0.35 × 0.29 × 0.12 mm
*Z* = 4	

### Data collection

**Table:**

Bruker SMART APEXII DUO CCD area-detector diffractometer	5389 independent reflections
Radiation source: fine-focus sealed tube	4508 reflections with *I* > 2σ(*I*)
Graphite monochromator	*R*_int_ = 0.026
φ and ω scans	θ_max_ = 32.5°, θ_min_ = 2.6°
Absorption correction: multi-scan (*SADABS*; Bruker, 2009)	*h* = −9→9
*T*_min_ = 0.449, *T*_max_ = 0.735	*k* = −18→18
20560 measured reflections	*l* = −30→28

### Refinement

**Table:**

Refinement on *F*^2^	Primary atom site location: structure-invariant direct methods
Least-squares matrix: full	Secondary atom site location: difference Fourier map
*R*[*F*^2^ > 2σ(*F*^2^)] = 0.026	Hydrogen site location: inferred from neighbouring sites
*wR*(*F*^2^) = 0.064	H-atom parameters constrained
*S* = 1.04	*w* = 1/[σ^2^(*F*_o_^2^) + (0.0284*P*)^2^ + 0.4844*P*] where *P* = (*F*_o_^2^ + 2*F*_c_^2^)/3
5389 reflections	(Δ/σ)_max_ = 0.002
200 parameters	Δρ_max_ = 0.50 e Å^−^^3^
0 restraints	Δρ_min_ = −0.25 e Å^−^^3^

### Special details

**Table:**

Experimental. The crystal was placed in the cold stream of an Oxford Cryosystems Cobra open-flow nitrogen cryostat (Cosier & Glazer, 1986) operating at 100.0 (1) K.
Geometry. All e.s.d.'s (except the e.s.d. in the dihedral angle between two l.s. planes) are estimated using the full covariance matrix. The cell e.s.d.'s are taken into account individually in the estimation of e.s.d.'s in distances, angles and torsion angles; correlations between e.s.d.'s in cell parameters are only used when they are defined by crystal symmetry. An approximate (isotropic) treatment of cell e.s.d.'s is used for estimating e.s.d.'s involving l.s. planes.
Refinement. Refinement of *F*^2^ against ALL reflections. The weighted *R*-factor *wR* and goodness of fit *S* are based on *F*^2^, conventional *R*-factors *R* are based on *F*, with *F* set to zero for negative *F*^2^. The threshold expression of *F*^2^ > σ(*F*^2^) is used only for calculating *R*-factors(gt) *etc*. and is not relevant to the choice of reflections for refinement. *R*-factors based on *F*^2^ are statistically about twice as large as those based on *F*, and *R*- factors based on ALL data will be even larger.

### Fractional atomic coordinates and isotropic or equivalent isotropic displacement parameters (Å^2^)

**Table:**

	*x*	*y*	*z*	*U*_iso_*/*U*_eq_	
Br1	0.04341 (2)	0.791941 (11)	0.448115 (6)	0.02747 (5)	
F1	−0.59388 (13)	1.16479 (7)	0.96048 (4)	0.02911 (17)	
O1	0.64441 (14)	0.86820 (8)	0.74360 (4)	0.02269 (17)	
N1	0.18952 (16)	0.95514 (8)	0.82286 (5)	0.01709 (17)	
N2	0.33651 (16)	0.94281 (8)	0.77529 (5)	0.01819 (18)	
C1	−0.12723 (19)	1.02079 (10)	0.90775 (6)	0.0197 (2)	
H1A	−0.0275	0.9644	0.9231	0.024*	
C2	−0.2902 (2)	1.05229 (10)	0.94636 (6)	0.0219 (2)	
H2A	−0.3024	1.0189	0.9883	0.026*	
C3	−0.43476 (19)	1.13377 (10)	0.92222 (6)	0.0198 (2)	
C4	−0.42365 (19)	1.18535 (9)	0.86209 (6)	0.0198 (2)	
H4A	−0.5265	1.2404	0.8468	0.024*	
C5	−0.25649 (19)	1.15407 (9)	0.82441 (6)	0.0182 (2)	
H5A	−0.2431	1.1896	0.7832	0.022*	
C6	−0.10798 (18)	1.07118 (9)	0.84631 (5)	0.01613 (19)	
C7	0.06512 (18)	1.03787 (9)	0.80585 (5)	0.01641 (19)	
C8	0.1174 (2)	1.09452 (10)	0.74299 (6)	0.0204 (2)	
H8A	0.1713	1.1694	0.7527	0.024*	
H8B	−0.0138	1.0972	0.7091	0.024*	
C9	0.30095 (19)	1.02199 (9)	0.71935 (6)	0.0182 (2)	
H9A	0.4379	1.0659	0.7171	0.022*	
C10	0.23654 (18)	0.96573 (9)	0.65297 (5)	0.01642 (19)	
C11	0.38005 (19)	0.96669 (9)	0.60390 (6)	0.0182 (2)	
H11A	0.5182	1.0028	0.6123	0.022*	
C12	0.3230 (2)	0.91522 (10)	0.54268 (6)	0.0193 (2)	
H12A	0.4217	0.9153	0.5095	0.023*	
C13	0.1196 (2)	0.86390 (9)	0.53106 (6)	0.0188 (2)	
C14	−0.02684 (19)	0.86179 (10)	0.57892 (6)	0.0204 (2)	
H14A	−0.1656	0.8263	0.5701	0.025*	
C15	0.03330 (19)	0.91257 (10)	0.64006 (6)	0.0193 (2)	
H15A	−0.0648	0.9111	0.6734	0.023*	
C16	0.50858 (18)	0.87211 (10)	0.78447 (6)	0.0179 (2)	
C17	0.5186 (2)	0.79944 (10)	0.84496 (6)	0.0226 (2)	
H17A	0.6488	0.7524	0.8465	0.034*	
H17B	0.3851	0.7546	0.8422	0.034*	
H17C	0.5285	0.8441	0.8855	0.034*	

### Atomic displacement parameters (Å^2^)

**Table:**

	*U*^11^	*U*^22^	*U*^33^	*U*^12^	*U*^13^	*U*^23^
Br1	0.03694 (8)	0.02603 (7)	0.01781 (6)	−0.00295 (5)	−0.00372 (4)	−0.00310 (5)
F1	0.0272 (4)	0.0295 (4)	0.0332 (4)	0.0103 (3)	0.0145 (3)	0.0057 (3)
O1	0.0178 (4)	0.0292 (4)	0.0216 (4)	−0.0003 (3)	0.0047 (3)	−0.0060 (3)
N1	0.0168 (4)	0.0200 (4)	0.0149 (4)	0.0013 (3)	0.0036 (3)	−0.0005 (3)
N2	0.0192 (4)	0.0214 (4)	0.0148 (4)	0.0026 (4)	0.0052 (3)	0.0007 (3)
C1	0.0195 (5)	0.0191 (5)	0.0207 (5)	0.0037 (4)	0.0036 (4)	0.0029 (4)
C2	0.0220 (5)	0.0230 (5)	0.0217 (5)	0.0041 (4)	0.0068 (4)	0.0055 (4)
C3	0.0177 (5)	0.0195 (5)	0.0230 (5)	0.0014 (4)	0.0060 (4)	−0.0011 (4)
C4	0.0199 (5)	0.0161 (5)	0.0228 (5)	0.0031 (4)	0.0001 (4)	−0.0005 (4)
C5	0.0214 (5)	0.0162 (5)	0.0165 (5)	0.0011 (4)	0.0003 (4)	0.0004 (4)
C6	0.0167 (5)	0.0150 (4)	0.0165 (5)	−0.0004 (4)	0.0009 (4)	−0.0020 (4)
C7	0.0177 (5)	0.0164 (5)	0.0151 (5)	−0.0006 (4)	0.0019 (4)	−0.0011 (4)
C8	0.0266 (6)	0.0180 (5)	0.0171 (5)	0.0026 (4)	0.0051 (4)	0.0010 (4)
C9	0.0207 (5)	0.0184 (5)	0.0160 (5)	−0.0013 (4)	0.0038 (4)	0.0004 (4)
C10	0.0178 (5)	0.0162 (5)	0.0157 (5)	−0.0008 (4)	0.0037 (4)	0.0015 (4)
C11	0.0182 (5)	0.0195 (5)	0.0174 (5)	−0.0039 (4)	0.0044 (4)	0.0008 (4)
C12	0.0229 (5)	0.0202 (5)	0.0155 (5)	−0.0018 (4)	0.0055 (4)	0.0018 (4)
C13	0.0229 (5)	0.0174 (5)	0.0155 (5)	−0.0004 (4)	−0.0007 (4)	0.0006 (4)
C14	0.0172 (5)	0.0206 (5)	0.0231 (5)	−0.0025 (4)	0.0008 (4)	0.0008 (4)
C15	0.0173 (5)	0.0215 (5)	0.0199 (5)	−0.0017 (4)	0.0055 (4)	0.0010 (4)
C16	0.0157 (5)	0.0204 (5)	0.0173 (5)	−0.0007 (4)	0.0003 (4)	−0.0046 (4)
C17	0.0241 (5)	0.0243 (5)	0.0191 (5)	0.0057 (4)	0.0008 (4)	0.0001 (4)

### Geometric parameters (Å, º)

**Table:**

Br1—C13	1.9003 (11)	C8—C9	1.5494 (16)
F1—C3	1.3622 (14)	C8—H8A	0.9900
O1—C16	1.2349 (14)	C8—H8B	0.9900
N1—C7	1.2919 (14)	C9—C10	1.5163 (16)
N1—N2	1.3947 (13)	C9—H9A	1.0000
N2—C16	1.3590 (15)	C10—C11	1.3945 (16)
N2—C9	1.4864 (15)	C10—C15	1.3987 (16)
C1—C2	1.3866 (16)	C11—C12	1.3940 (16)
C1—C6	1.4013 (16)	C11—H11A	0.9500
C1—H1A	0.9500	C12—C13	1.3867 (17)
C2—C3	1.3853 (16)	C12—H12A	0.9500
C2—H2A	0.9500	C13—C14	1.3887 (17)
C3—C4	1.3760 (17)	C14—C15	1.3916 (17)
C4—C5	1.3942 (17)	C14—H14A	0.9500
C4—H4A	0.9500	C15—H15A	0.9500
C5—C6	1.4008 (15)	C16—C17	1.5070 (17)
C5—H5A	0.9500	C17—H17A	0.9800
C6—C7	1.4653 (16)	C17—H17B	0.9800
C7—C8	1.5116 (16)	C17—H17C	0.9800
			
C7—N1—N2	107.94 (9)	N2—C9—C8	101.04 (9)
C16—N2—N1	121.60 (9)	C10—C9—C8	114.33 (10)
C16—N2—C9	124.40 (9)	N2—C9—H9A	109.8
N1—N2—C9	113.63 (9)	C10—C9—H9A	109.8
C2—C1—C6	120.72 (11)	C8—C9—H9A	109.8
C2—C1—H1A	119.6	C11—C10—C15	119.19 (10)
C6—C1—H1A	119.6	C11—C10—C9	120.12 (10)
C3—C2—C1	118.28 (11)	C15—C10—C9	120.69 (10)
C3—C2—H2A	120.9	C12—C11—C10	120.71 (11)
C1—C2—H2A	120.9	C12—C11—H11A	119.6
F1—C3—C4	118.69 (10)	C10—C11—H11A	119.6
F1—C3—C2	118.06 (11)	C13—C12—C11	118.83 (11)
C4—C3—C2	123.25 (11)	C13—C12—H12A	120.6
C3—C4—C5	117.78 (11)	C11—C12—H12A	120.6
C3—C4—H4A	121.1	C12—C13—C14	121.75 (11)
C5—C4—H4A	121.1	C12—C13—Br1	118.87 (9)
C4—C5—C6	121.09 (11)	C14—C13—Br1	119.37 (9)
C4—C5—H5A	119.5	C13—C14—C15	118.78 (11)
C6—C5—H5A	119.5	C13—C14—H14A	120.6
C5—C6—C1	118.87 (10)	C15—C14—H14A	120.6
C5—C6—C7	120.64 (10)	C14—C15—C10	120.73 (11)
C1—C6—C7	120.49 (10)	C14—C15—H15A	119.6
N1—C7—C6	120.77 (10)	C10—C15—H15A	119.6
N1—C7—C8	114.27 (10)	O1—C16—N2	120.09 (11)
C6—C7—C8	124.95 (10)	O1—C16—C17	123.28 (11)
C7—C8—C9	102.84 (9)	N2—C16—C17	116.61 (10)
C7—C8—H8A	111.2	C16—C17—H17A	109.5
C9—C8—H8A	111.2	C16—C17—H17B	109.5
C7—C8—H8B	111.2	H17A—C17—H17B	109.5
C9—C8—H8B	111.2	C16—C17—H17C	109.5
H8A—C8—H8B	109.1	H17A—C17—H17C	109.5
N2—C9—C10	111.65 (9)	H17B—C17—H17C	109.5
			
C7—N1—N2—C16	170.61 (10)	C16—N2—C9—C8	−168.23 (11)
C7—N1—N2—C9	−2.78 (13)	N1—N2—C9—C8	4.94 (12)
C6—C1—C2—C3	0.80 (19)	C7—C8—C9—N2	−4.85 (11)
C1—C2—C3—F1	−179.69 (11)	C7—C8—C9—C10	115.20 (10)
C1—C2—C3—C4	−0.44 (19)	N2—C9—C10—C11	−112.65 (12)
F1—C3—C4—C5	178.57 (10)	C8—C9—C10—C11	133.42 (11)
C2—C3—C4—C5	−0.67 (18)	N2—C9—C10—C15	67.46 (13)
C3—C4—C5—C6	1.44 (17)	C8—C9—C10—C15	−46.47 (15)
C4—C5—C6—C1	−1.10 (17)	C15—C10—C11—C12	−0.17 (17)
C4—C5—C6—C7	179.11 (11)	C9—C10—C11—C12	179.94 (10)
C2—C1—C6—C5	−0.05 (18)	C10—C11—C12—C13	0.69 (18)
C2—C1—C6—C7	179.74 (11)	C11—C12—C13—C14	−0.57 (18)
N2—N1—C7—C6	179.71 (10)	C11—C12—C13—Br1	−179.12 (9)
N2—N1—C7—C8	−0.93 (13)	C12—C13—C14—C15	−0.08 (18)
C5—C6—C7—N1	−173.72 (11)	Br1—C13—C14—C15	178.47 (9)
C1—C6—C7—N1	6.49 (17)	C13—C14—C15—C10	0.61 (18)
C5—C6—C7—C8	6.99 (17)	C11—C10—C15—C14	−0.49 (17)
C1—C6—C7—C8	−172.79 (11)	C9—C10—C15—C14	179.39 (11)
N1—C7—C8—C9	3.93 (13)	N1—N2—C16—O1	−175.15 (10)
C6—C7—C8—C9	−176.74 (10)	C9—N2—C16—O1	−2.50 (17)
C16—N2—C9—C10	69.83 (14)	N1—N2—C16—C17	6.25 (16)
N1—N2—C9—C10	−117.00 (10)	C9—N2—C16—C17	178.90 (10)

### Hydrogen-bond geometry (Å, º)

**Table:**

*D*—H···*A*	*D*—H	H···*A*	*D*···*A*	*D*—H···*A*
C4—H4*A*···O1^i^	0.95	2.45	3.2772 (15)	146
C14—H14*A*···F1^ii^	0.95	2.50	3.3806 (15)	153
C15—H15*A*···O1^iii^	0.95	2.45	3.3800 (15)	166

Symmetry codes: (i) −*x*, *y*+1/2, −*z*+3/2; (ii) −*x*−1, *y*−1/2, −*z*+3/2; (iii) *x*−1, *y*, *z*.
